# Correction: Ahmed et al. A Robust Deep Feature Extraction Method for Human Activity Recognition Using a Wavelet Based Spectral Visualisation Technique. *Sensors* 2024, *24*, 4343

**DOI:** 10.3390/s25133966

**Published:** 2025-06-26

**Authors:** Nadeem Ahmed, Md Obaydullah Al Numan, Raihan Kabir, Md Rashedul Islam, Yutaka Watanobe

**Affiliations:** 1 Department of Computer Science and Engineering, University of Asia Pacific, Dhaka 1216, Bangladesh; nadeem@uap-bd.edu; 2 Department of Computer Science and Engineering, University of Aizu, Aizu-Wakamatsu 965-8580, Japan; obaydullahhasib.cse@gmail.com (M.O.A.N.); raihan.kabir.cse@gmail.com (R.K.); yutaka@u-aizu.ac.jp (Y.W.)

There was an error in the original publication [1]. While the authors were working on the further implementation of this research, they noticed this error of reusing the contents from Reference 57.

A correction has been made to Section 4.1, Paragraph 2. The sentence “The dataset encompasses a comprehensive set of 19 Activities of Daily Living (ADL) performed by individuals across different age groups, ranging from ages 19 to 50.” has been updated to “The dataset encompasses a comprehensive set of 19 Activities of Daily Living (ADL) performed by two age groups. Elderly people group was comprised of 15 individuals (8 males aged 60–71 and 7 females aged 62–75) whereas young adults’ group was comprised of 23 participants (both males and females aged 19–30).”.

The authors state that the scientific conclusions are unaffected. This correction was approved by the Academic Editor. The original publication has also been updated.
